# Supplementary material for: Oocyst Shedding Dynamics in Children with Cryptosporidiosis: a Prospective Clinical Case Series in Ethiopia
Source: Microbiol Spectr. 2022 Jun 14;10(4):e02741-21. doi: 10.1128/spectrum.02741-21 (PMC9430463; doi:10.1128/spectrum.02741-21)

Figure S1. *Cryptosporidium* shedding over time, results for individual participants.

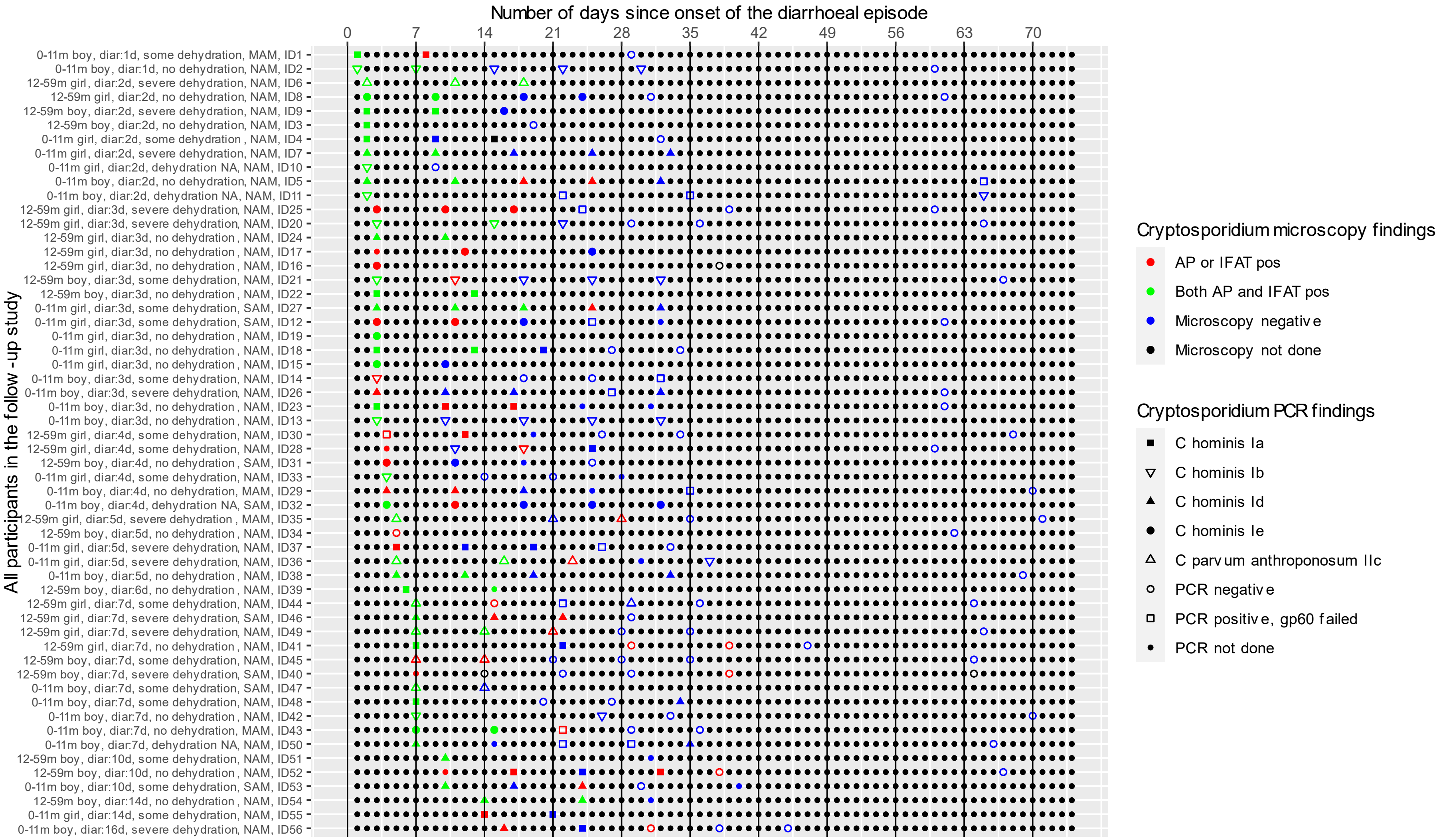

Note: For *Cryptosporidium* qPCR quantity and subtyping results, see **Supplementary dataset**.

Figure S2. Visual comparison and selection of time-to-event parametric models

The time-to-event graphs in Figure 1 in the main manuscript represent the aggregate interval-censored time-to-event data, overall, and stratified by subgroups. The graphs are based on the non-parametric maximum likelihood estimator (NPLME; the Turnbull estimator) and resembles a Kaplan Meier plot but is extended to allow for interval censored data. To our knowledge, there are no well-established methods to obtain confidence intervals for time-to-event duration estimates, using non-parametric or semi-parametric models. We therefore fitted several parametric models. Parametric models have the added advantage that we were able to adjust for the (a priori-assumed confounding factors of) age and sex. As there are no generally approved formal methods for choosing between time-to-event parametric models for interval censored data, we used the diag_baseline function from the icenReg R package [1] to compare various parametric models visually, with a semi-parametric Turnbull model as a baseline for assessing model fit; all models were adjusted for sex and age (in months):

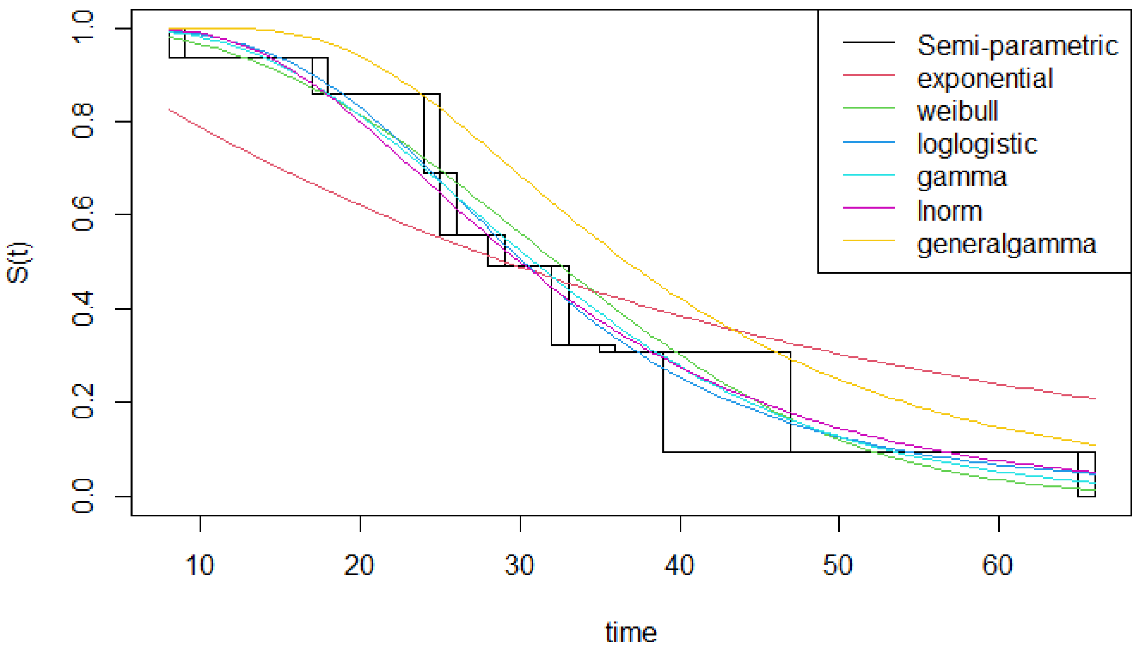

Although visual diagnostics are somewhat subjective, we see that most of the the parametric distributions show no systematic deviation from the semi-parametric baseline model, with the notable exception of the exponential and generalized gamma distributions. This implies that various parametric model familes may do a reasonable job of describing the underlying distribution. We opted for the commonly used log-logistic model, which allowed us to estimate median shedding duration with 95% confidence intervals and, also, to compare time-to-event curves between key subgroups of children by estimation of the odds ratio (also with 95% confidence intervals).

1. Anderson-Bergman C. icenReg: regression models for interval censored data in R. Journal of Statistical Software. 2017;81(1):1-23.

Supplementary table S1. Estimated *Cryptosporidium* quantity over time. Estimated by the overall generalized additive mixed model.

Weeks since onset of diarrhoea	*Cryptosporidium* DNA quantity (log10 copies/g)	95% confidence interval
0	7.7	6.6 to 8.7
1	6.6	5.6 to 7.6
2	5.5	4.5 to 6.5
3	4.5	3.5 to 5.5
4	3.6	2.6 to 4.6
5	2.8	1.8 to 3.8
6	2.2	1.2 to 3.2
7	1.6	0.58 to 2.7
8	1.2	0.02 to 2.3

Figure S3. Plot of difference in shedding quantity over time, compared between subgroups.

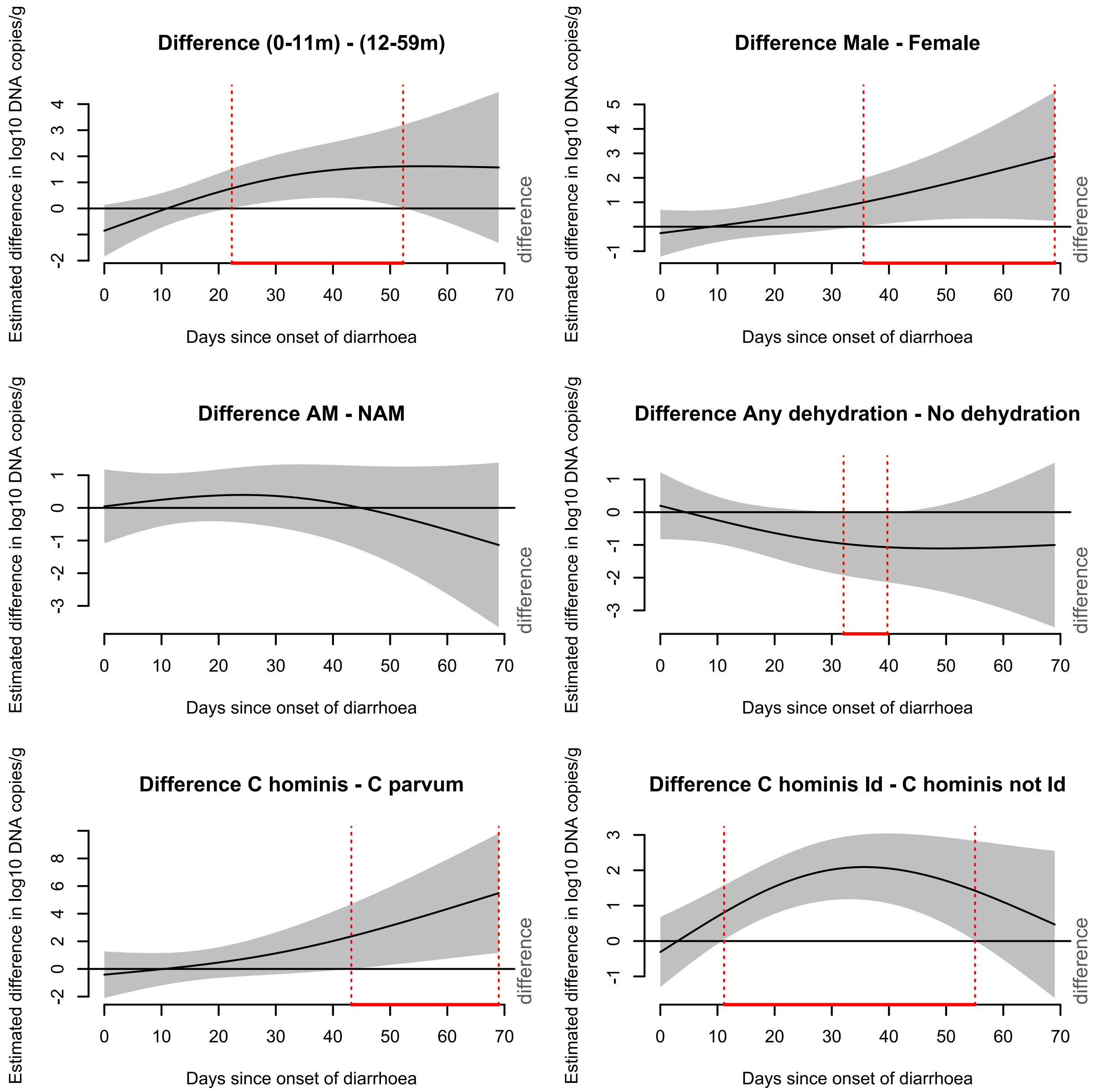

Note: These plots were made with the plot_diff function from the R package itsadug (https://CRAN.R-project.org/package=itsadug)

Figure S4. Plot of GAMM model used to predict shedding duration from overall drop in *Cryptosporidium* DNA quantity over time. Vertical dashed line represents the detection limit of the qPCR assay, and the green shaded ribbon represents the 95% confidence interval for the model predicted days since onset of diarrhoea.

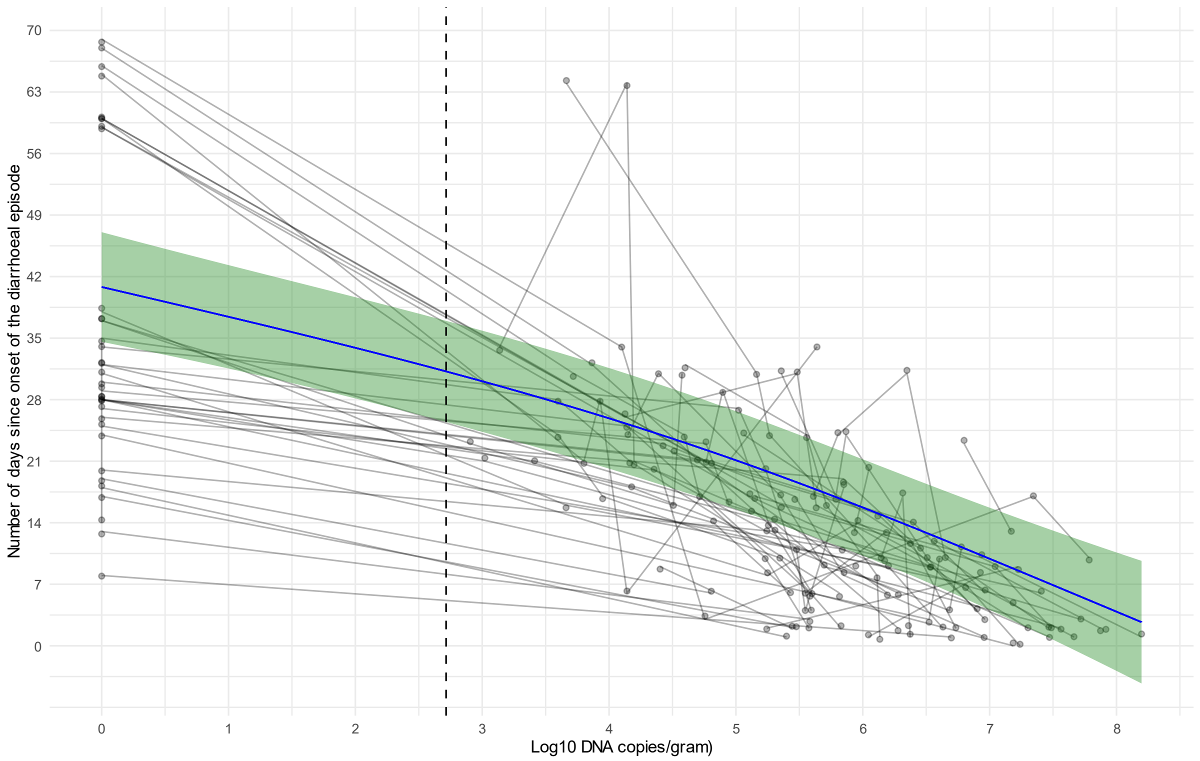

Supplement: Supplemental file 2 — Fig. S1 to S4 and Table S1. Download spectrum.02741-21-s0002.pdf, PDF file, 1.1 MB [file spectrum.02741-21-s0002.pdf]
